# Decreased voluntary alcohol intake and ventral striatal epigenetic and transcriptional remodeling in male Acss2 KO mice

**DOI:** 10.1016/j.neuropharm.2024.110258

**Published:** 2024-12-09

**Authors:** Gabor Egervari, Greg Donahue, Natalia A.Quijano Cardé, Desi C. Alexander, Connor Hogan, Jessica K. Shaw, Erica M. Periandri, Vanessa Fleites, Mariella De Biasi, Shelley L. Berger

**Affiliations:** a Department of Genetics, Washington University School of Medicine, St. Louis, MO, USA; b Department of Biochemistry and Molecular Biophysics, Washington University, School of Medicine, St. Louis, MO, USA; c Epigenetics Institute, Perelman School of Medicine, University of Pennsylvania, Philadelphia, PA, USA; d Department of Cell and Developmental Biology, Perelman School of Medicine, University of Pennsylvania, Philadelphia, PA, USA; e Department of Psychiatry, Perelman School of Medicine, University of Pennsylvania, Philadelphia, PA, USA

## Abstract

Metabolic-epigenetic interactions are emerging as key pathways in regulating alcohol-related transcriptional changes in the brain. Recently, we have shown that this is mediated by the metabolic enzyme Acetyl-CoA synthetase 2 (Acss2), which is nuclear and chromatin-bound in neurons. Mice lacking ACSS2 fail to deposit alcohol-derived acetate onto histones in the brain and show no conditioned place preference for ethanol reward. Here, we further explored the role of this pathway during voluntary alcohol intake. We found that Acss2 KO mice consume significantly less alcohol in a model of binge drinking, an effect primarily driven by males. Genome-wide transcriptional profiling of 7 key brain regions implicated in alcohol and drug use revealed that, following drinking, Acss2 KO mice exhibit blunted gene expression in the ventral striatum. Similarly to the behavioral differences, transcriptional dysregulation was more pronounced in male mice. Further, we found that the gene expression changes were associated with depletion of ventral striatal histone acetylation (H3K27ac) in Acss2 KO mice compared to WT. Taken together, our data suggest that ACSS2 plays an important role in orchestrating ventral striatal epigenetic and transcriptional changes during voluntary alcohol drinking, especially in males. Consequently, targeting this pathway could be a promising new therapeutic avenue.

## 1. Introduction

Substance use disorders, including alcohol use disorder (AUD), impose a tremendous burden on society as conventional pharmacotherapies lack substantial and durable efficacy (Mason, 2017; Johnson, 2008; Witkiewitz et al., 2019). Epigenetic mechanisms (Berger, 2007), such as histone acetylation, which dynamically regulate gene expression were strongly linked both to acute alcohol exposure and to AUD (Berkel and Pandey, 2017; Mons and Beracochea, 2016; Robison and Nestler, 2011; Zakhari, 2013; Palmisano and Pandey, 2017; Finegersh et al., 2015; Finegersh and Homanics, 2014; Mahnke et al., 2017; Egervari et al., 2021). Therefore, emerging new approaches based on epigenetic mechanisms hold considerable promise for AUD and psychiatric illness in general (Karsli-Ceppioglu, 2016; Moos et al., 2016; Savarese and Lasek, 2018; Egervari et al., 2017a, 2017b, 2018, 2020a). For example, histone deacetylase inhibitors were shown to decrease binge-like alcohol drinking in mice (Warnault et al., 2013).

Epigenetic mechanisms are strongly influenced by metabolic processes (Li et al., 2018; Egervari et al., 2020b). In fact, metabolism of ethanol itself has been proposed to contribute to alcohol-induced epigenetic alterations (Zakhari, 2013). Until recently, this has been mostly attributed to changes in available substrates and cofactors (Zakhari, 2013; Lu et al., 2000; Medici and Halsted, 2013), increases in the NADH/NAD ratio (Zakhari, 2013; Imai et al., 2000), or reactive oxygen species production (Ziech et al., 2011). Importantly, however, alcohol metabolism gives rise to surges of acetate in the systemic circulation following alcohol exposure (Sarkola et al., 2002), which can directly affect brain function (Jiang et al., 2013).

Recently, we described a novel, direct and rapid mechanism for alcohol-driven brain histone acetylation governed by a metabolic enzyme moonlighting in neuronal nuclei. This enzyme, ACSS2 (Acetyl-CoA Synthetase 2), is bound to promoters of key neuronal genes in the dorsal hippocampus (a brain region involved in learning and strongly affected by alcohol (Taffe et al., 2010)), and leads to increased histone acetylation and transcription upon alcohol exposure. Strikingly, we showed that this is driven by direct and rapid incorporation of alcohol-derived acetate into histone acetylation, which has been previously shown only in the liver (Kriss et al., 2018). The resulting gene expression changes guide associative memory formation of alcohol-related environmental cues (Mews et al., 2019). This is important because drug-associated memories can drive craving and relapse even after protracted periods of abstinence and thus play a central role in driving alcohol consumption and the development of AUD (Barak et al., 2013; Robinson and Berridge, 1993; Milton, 2013). Of note, *ACSS2* polymorphisms have recently been linked to AUD in humans (Ribeiro et al., 2017).

Importantly, our prior research utilized models of passive exposure to ethanol. While this strategy allowed us to directly track acetate incorporation into the brain epigenome, it did not address important questions about the relevance of this pathway in models of voluntary alcohol drinking that better approximate patterns of human alcohol consumption. Therefore, we now assessed the effect of genetic Acss2 inhibition on voluntary alcohol intake and simultaneously assayed the epigenetic and transcriptional changes that accompany voluntary alcohol consumption in key brain regions linked to AUD.

## 2. Materials and methods

### 2.1. Animals

Animal use and all experiments performed were approved by the Institutional Animal Care and Use Committee (IACUC protocols 804849 and 805052). All personnel involved have been adequately trained and are qualified according to the Animal Welfare Act (AWA) and the Public Health Service (PHS) policy. To prevent genetic drift, Acss2 knockout (KO, created on C57BL/6J background as originally described in (Alexander et al., 2022)) and corresponding WT controls were generated through homozygous crosses of F1 progeny from heterozygote breeding cages. Mice were housed on a 12h/12h light/dark, with food and water provided ad libitum. All behavioral experiments were conducted during the first 4 h of the dark cycle to reduce time-of-day effects.

### 2.2. Drinking-in-the-dark paradigm

Single-housed adult mice (approximately 3–5 months old) of both sexes were allowed to voluntarily consume ethanol in an adapted version of the drinking-in-the-dark (DID) paradigm, an extensively used rodent model of binge-like drinking (Rhodes et al., 2005). During the habituation period, mice were acclimated to having two bottles (2BC) in the housing cage. Following the 1-week habituation period, ethanol intake was evaluated in the DID-2BC paradigm. For this, mice had access to 15% (v/v) ethanol and water for 2 h on days 1–3 and 4 h on day 4. The position of the bottles was alternated daily to avoid side preference. On the second week of DID, mice had access to 15% ethanol in a similar schedule, but water was not available during the drinking session (DID-1B).

All drinking sessions started 1.0–1.5 h into the dark phase of the light cycle. Bottles were weighed to the nearest hundredth at the beginning and end of each DID session to determine fluid consumption. Mice were weighed at the beginning of each DID session to determine ethanol exposure (g/kg), calculated using r15_ethanol = 0.97897 g/mL and r100_ethanol = 0.789 g/mL. Ethanol preference was calculated as the ratio of ethanol to total fluid intake (mL ethanol solution/mL total fluid).

Animals were sacrificed following the final DID-1B drinking session via decapitation. Brains were extracted and flash frozen, and subsequently stored at −80 °C.

### 2.3. Blood ethanol concentration (BEC)

Mice were sacrificed immediately following the final drinking session and blood was collected. Serum ethanol concentration was measured using the Ethanol Assay Kit from Sigma-Aldrich^®^ (Cat No. MAK076). Samples were prepared according to manufacturer protocol. Colorimetric readout was done using a BioTek^®^ Synergy™ H1 microplate reader for absorbance at 570 nm. Data are normalized to female WT mice to eliminate batch effects. For technical reasons, we were not able to collect data for three mice.

### 2.4. Ethanol clearance assay

Mice were injected intraperitoneally with 2 g/kg EtOH (20% v/v) in saline. Blood was collected under general anesthesia (isoflurane in O2) 30 min (retro-orbital via heparinized capillary tubes) and 240 min (trunk blood) after injection. Mice recovered with thermal support between timepoints. Blood was stored in heparinized collection tubes on ice until centrifugation (4000 rpm at 4 °C). Resulting plasma was aliquoted and stored at −80 °C until analysis. Ethanol concentration across both timepoints was quantified in duplicates using a commercially available ethanol assay (ab6543, Abcam, Cambridge, MA) according to manufacturer’s instructions. Plasma samples were diluted 1:100 and 15 μL was loaded per well, and ethanol concentration was determined by optical density (OD 570 nm) using SpectraMax 190 colorimetric plate reader (Molecular Devices, San Jose, CA) and SoftMax Pro 5 software (Molecular Devices).

### 2.5. Sucrose and quinine drinking

Mice were individually housed and acclimated to drinking conditions for 1 week. Drinking fluids were provided in conical tubes fitted with rubber stoppers and metal sippers as previously described. Mice were presented with one bottle containing water and another bottle containing either 1% sucrose or 0.03 μM quinine. Each tastant was presented for four consecutive days each, with a “washout” day (water only) in between tastants. Bottles were weighed each day and sides alternated; a cage containing bottles but no mice was used to correct for changes in bottle weight due to leakage. Preference was calculated as Intake_tastant_/Intake_tastant + water_. Preference for each tastant was averaged over the last two days of exposure to account for side preferences and compared between groups.

### 2.6. RNA-sequencing

To measure transcriptional responses across different brain regions, we have dissected 10–20 mg tissue using a 1 mm brain matrix (BSMAS001–1, Zivic Instruments) and 1 mm sample corers (18035–01, Fine Science Technologies) from the following brain regions of both male and female WT and Acss2 KO mice: dorsal and ventral hippocampus, dorsal and ventral striatum, amygdala, cortex, and prefrontal (prelimbic/infralimbic) cortex. Total RNA was extracted using Trizol-chloroform. Total RNA quality was assessed on the Bioanalyzer platform using the RNA 6000 Nano assay (Agilent). mRNA was isolated from 300 ng total RNA using the NEBNext^®^ Poly(A) mRNA Magnetic Isolation Module (E7490L), and libraries were prepared using the NEBNext^®^ UltraTM II RNA Library Prep Kit for Illumina^®^ (E7770).

Sequencing data were aligned to mouse genome assembly GRCm38/mm10 using STAR v2.5.2a with command-line parameters –outFilterType BySJout –outFilterMultimapNmax 20 –alignSJoverhangMin 8 –alignSJDBoverhangMin 1 –outFilterMismatchNmax 999 –alignIntronMin 20 –alignIntronMax 1000000 –alignMatesGapMax 1000000 –readFilesCommand zcat –outFilterMismatchNoverReadLmax 0.04. The STAR index was constructed GENCODE M25 annotation (ENSEMBL 100 assembly). Data were filtered for poor mapping tags using samtools v1.1 subcommand view -bS -q 255. Technical repeat samples from two flowcells were combined using samtools merge and aligned tags were sorted by chromosome position and indexed using samtools sort and index, respectively. HTseq v0.6.1 was used to quantify tag counts over exons with command-line parameters -f bam -r pos -s reverse -t exon -i gene_id –additional-attr = gene_name. Differentially expressed genes (DEGs) between Acss2 KO and WT samples were detected separately in each tissue using DESeq2 in R v4.0.2 with the FDR controlled at 0.1.

Heatmaps were generated using R library pheatmap and PCA was rendered from the full data matrix with zero rows removed, using ggplot2. Volcano plots were also generated using ggplot2. Gene Ontology analysis was performed using DAVID with the FDR controlled at 0.1. RNA-seq data were visualized on the UCSC Genome Browser; tracks were created using DeepTools bamCoverage v3.4.3 with command-line parameter –scaleFactor set to the reciprocal DESeq2 size factor for that sample. Finally, bigWigs from biological repeats were combined using wiggletools (“mean” function) and then rendered from bedGraph to bigWig using UCSC Genome Browser tools’ bedGraphToBigWig.

### 2.7. H3K27ac ChIP-seq

Approximately 20 mg of ventral striatal tissue from each mouse was minced on ice, cross-linked with 1% formaldehyde for 10 min and quenched with 125 mM glycine for 5 min. Nuclei were prepared by dounce homogenization of cross-linked tissue in nuclei isolation buffer (50 mM Tris-HCl at pH 7.5, 25 mM KCl, 5 mM MgCl2, 0.25 M sucrose) with freshly added 1x Halt protease and phosphatase inhibitor cocktail (Thermo Scientific, PI78445) and 10 mM sodium butyrate. Nuclei were lysed in nuclei lysis buffer (10 mM Tris-HCl at pH 8.0, 100 mM NaCl, 1 mM EDTA, 0.5 mM EGTA, 0.1% sodium deoxycholate, 0.5% *N*-lauroylsarcosine) with freshly added 1x Halt protease and phosphatase inhibitor cocktail and 10 mM sodium butyrate, and chromatin was sheared using a Covaris S220 sonicator to approximately 250 bp in size. Equal aliquots of sonicated chromatin were used per immunoprecipitation reaction with 4 μl H3K27ac antibody (Abcam; 4729) preconjugated to Protein G Dynabeads (Life Technologies). Ten percent of the chromatin was saved as input DNA. ChIP reactions were incubated overnight at 4 °C with rotation and washed three times in wash buffer. Immuno-precipitated DNA was eluted from the beads, reversed cross-linked and purified together with the input DNA. Exactly 10 ng DNA (either ChIP or input) was used to construct sequencing libraries using the NEBNext Ultra II DNA library preparation kit for Illumina (New England Biolabs; NEB). Libraries were multiplexed using NEBNext Multiplex Oligos for Illumina (dual index primers) and paired-end sequenced (42 bp) on the NextSeq 500 platform (Illumina) in accordance with the manufacturer’s protocol.

Sequencing data were aligned to mouse genome assembly GRCm38/mm10 using bowtie2 v2.3.4.3 with command-line parameter –local. Data were filtered for poor mapping tags using samtools v1.1 subcommand view -bS -q 5 -f 2. Data were sorted by tag name using samtools sort -n and PCR duplicates were removed using PICARD MarkDuplicates v 2.21.3-SNAPSHOT with command-line parameters REMOVE_DUPLICATES = True ASSUME_SORT_ORDER = queryname. Aligned, deduplicated tags were sorted by chromosome position and indexed using samtools sort and index, respectively. RGT-THOR v0.13.2 was used to detect differentially enriched peaks using input samples to control for sonication efficiency artifacts at regions of open chromatin. RPM-adjusted counts over each differential peak were then assessed using a python v3 script. Peaks were associated to the nearest gene using HOMER v4.6 annotatePeaks.pl and reported annotation was used to construct genomic distribution pie charts in Supplementary Figs. 3A–B.

Peaks were filtered for a 4x alteration in Acss2 KO vs WT and the losses were scanned for motif enrichment using HOMER findMotifsGenome.pl using command-line parameters -size 300 -mask and using the 4x gained peaks as background. The reverse scan was also carried out for the gained peaks, using the losses as background. ChIP-seq data were visualized using UCSC Genome Browser; tracks were created using DeepTools bamCompare v3.4.3 with command-line parameters -bs 1 –effectiveGenomeSize 2652783500 and setting input to the background using parameter -b2. Gene Ontology analysis was performed using DAVID with the FDR controlled at 0.1. Boxed violin plots relating H3K27ac changes to gene expression changes were created using the ggplot2 library in R v4.0.2. Heatmaps of lost or gained H3K27ac peaks reflect a 2 kb window around the peak center and were created using python v3 scripts (vector quantitation and visualization using the PIL library).

## 3. Results

### 3.1. Loss of Acss2 reduces voluntary alcohol intake, especially in males

**Fig. 1.**
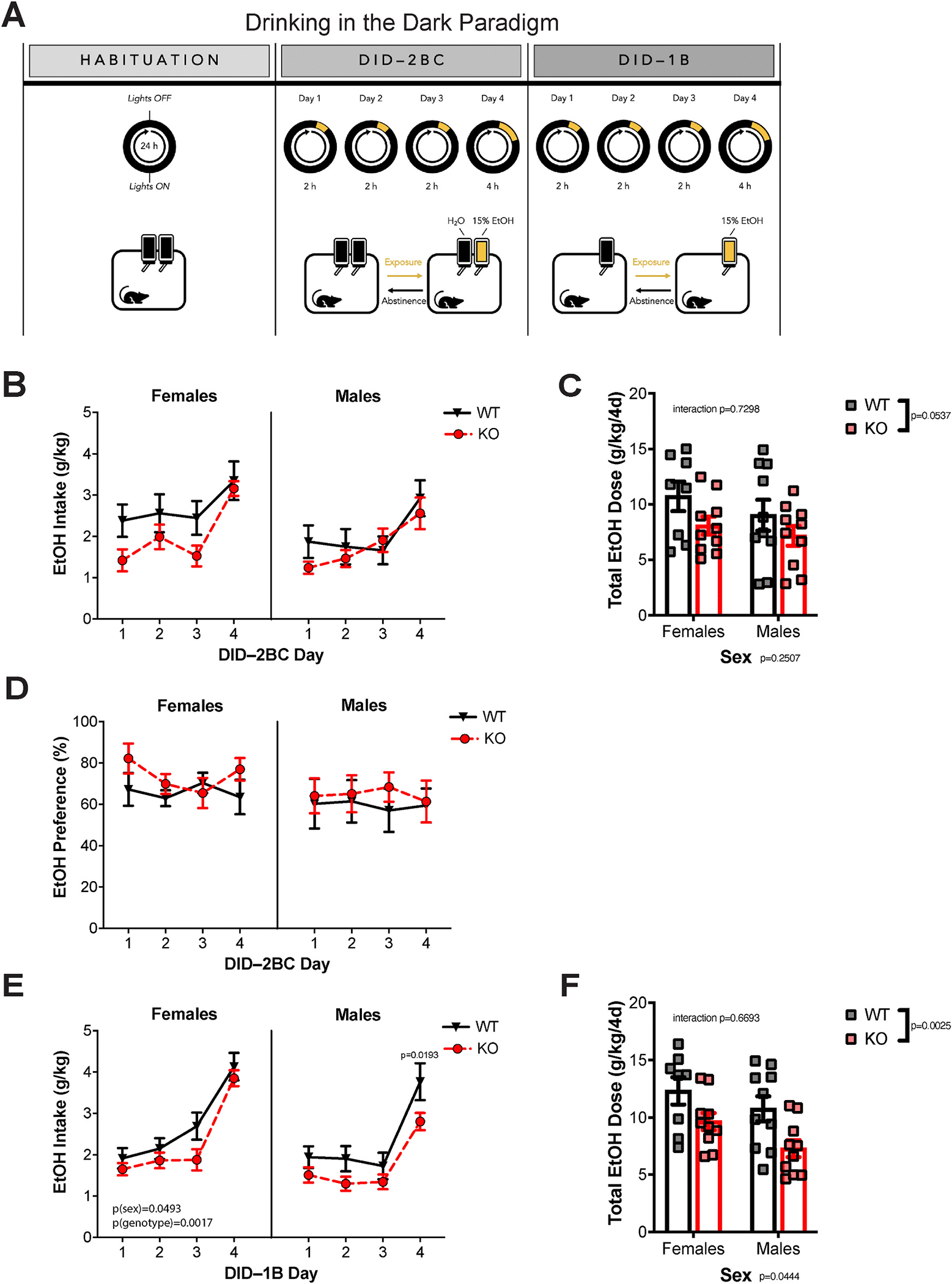
Decreased voluntary alcohol intake in Acss2 KO mice. (A) Schematic depicting the drinking-in-the-dark (DID) behavioral paradigm. Following habituation, mice had access to 15% (v/v) ethanol and water for 2–4 h during 4 days (2BC phase), then 15% (v/v) ethanol for 2–4 h during 4 days (1B phase). (B) Ethanol intake (g/kg) during the 2BC phase in female and male WT and Acss2 KO mice. (C) Cumulative ethanol intake during the 2BC phase of the DID paradigm. Two-way ANOVA revealed no significant effect of genotype (p = 0.0537). (D) Ethanol preference (%) in female and male WT and Acss2 KO mice. (E) Ethanol intake (g/kg) during the 1B phase in female and male WT and ACSS2 KO mice. During the 1B phase, three-way ANOVA revealed significant effects of both sex (F_1,34_ = 4.159, p = 0.0493) and genotype (F_1,34_ = 11.57, p = 0.0017). Post-hoc comparisons showed a significant decrease of alcohol intake on the final day of DID–1B in male mice (p-adj. = 0.0193), whereas no statistically significant differences were observed in female mice. (F) Cumulative ethanol intake during the 1B phase of the DID paradigm. Two-way ANOVA revealed significant effect of genotype (p = 0.0025), with post-hoc comparisons showing significantly decreased ethanol intake in male KOs (p-adj. = 0.0216). Symbols/columns and error bars represent the average ± SEM. Sample sizes are as follows: female WT (n = 8), female KO (n = 10), male WT (n = 10), male KO (n = 10). 2BC, 2 bottle choice; 1B, 1 bottle; KO, ACSS2 KO.

We evaluated binge-like ethanol drinking behavior in a modified version of the DID paradigm (Fig. 1A). DID is a widely used model of binge drinking in mice in which animals typically consume enough alcohol to reach high blood ethanol concentrations (BECs)(Rhodes et al., 2005). We used a modified version of DID in which male and female mice had access to 15% (v/v) ethanol and water during days 1–4 (two bottle choice, 2BC phase), and only 15% (v/v) ethanol during phase 1B (one bottle; Fig. 1A). To investigate the role of ACSS2 during drinking behavior, we used a constitutive Acss2 knockout (KO) line we recently developed on C57BL/6J background (Alexander et al., 2022). Importantly, loss of Acss2 is well tolerated and this genetically engineered mouse exhibits no overt behavioral abnormalities or transcriptional and epigenetic changes at baseline.

Acss2 KO had no effect on daily ethanol intake (two-way ANOVA genotype effect F_1,18_ = 1.268, p = 0.2749) or preference (two-way ANOVA genotype effect F_1,18_ = 0.0058, p = 0.9401) during the 2BC phase of the DID model (Fig. 1B–D). While cumulative ethanol intake tended to be lower in Acss2 KO mice during the 2BC phase (Fig. 1D), this effect did not reach statistical significance (two-way ANOVA genotype effect F_1,34_ = 3.995, p = 0.0537). During the 1B phase, however, we found that Acss2 KO decreased alcohol drinking in a sex-specific manner (Fig. 1E). Three-way ANOVA revealed significant effects of both sex (F_1,34_ = 4.159, p = 0.0493) and genotype (F_1,34_ = 11.57, p = 0.0017): Acss2 KO mice consumed less alcohol compared to WT controls, and this effect was primarily driven by males. Post-hoc comparisons showed a significant decrease of alcohol intake on the final day of DID in male mice (Fig. 1D, p-adj. = 0.0193), while no statistically significant differences were observed in female mice. Cumulative ethanol intake during the 1B phase (Fig. 1F) was significantly lower in Acss2 KO mice (two-way ANOVA genotype effect F_1,34_ = 10.67, p = 0.0025). Post-hoc comparisons revealed a significant decrease in males (p-adj = 0.0216) but not in females (p-adj. = 0.1158).

Mice were sacrificed immediately following the final DID-1B session and blood was collected to measure BEC. Intriguingly, we found that deletion of Acss2 had a sex-specific effect. While no difference was observed in male mice, female Acss2 KO mice had significantly higher BECs (student’s t-test, p = 0.0287) compared to WT controls (Supplementary Fig. 1A). To address potential differences in ethanol clearance as a confounding factor, we performed an ethanol clearance assay in both WT and Acss2 KO mice by measuring BEC both 30 min and 240 min following i.p. injection of 2 g/kg EtOH (Supplementary Fig. 1B). Mixed model ANOVA (between subjects: genotype; within subjects: time points) revealed that, as expected, BECs were significantly lower at 240 min compared to 30 min (F_1,18_ = 2642.25, p < 0.001). There was no significant interaction between genotype and time (F_1,18_ = 0.249, p = 0.624) and no main effect of genotype (F_1,18_ = 0.025, p = 0.876), indicating that Acss2 KO did not affect ethanol clearance.

Next, to address potential confounding effects of Acss2 KO on sweet and bitter taste perception, a separate cohort of mice was tested for sucrose and quinine consumption. We found no difference in the preference for sucrose (one-way ANOVA, F_1,18_ = 0.159, p = 0.695) or quinine (one-way ANOVA, F_1,18_ = 0.125, p = 0.728) in Acss2 KO mice compared to WT controls (Supplementary Figs. 1C and D). After stratifying for sex (Supplementary Figs. 1E and F), there were no differences for sweet perception in either males or females (two-way ANOVA genotype effect F_1,16_ = 0.219, p = 0.646), but female KOs had a greater preference for quinine compared to WTs (two-way ANOVA sex*genotype interaction F_1,16_ = 6.758, p < 0.05; post-hoc Sidak p < 0.05 in females). No differences in quinine preference were observed in males, suggesting that the decreased alcohol consumption in male Acss2 KO mice was not driven by altered bitter taste perception.

### 3.2. Blunted alcohol drinking-related transcriptional states in the ventral striatum of Acss2 KO mice compared to WT

**Fig. 2.**
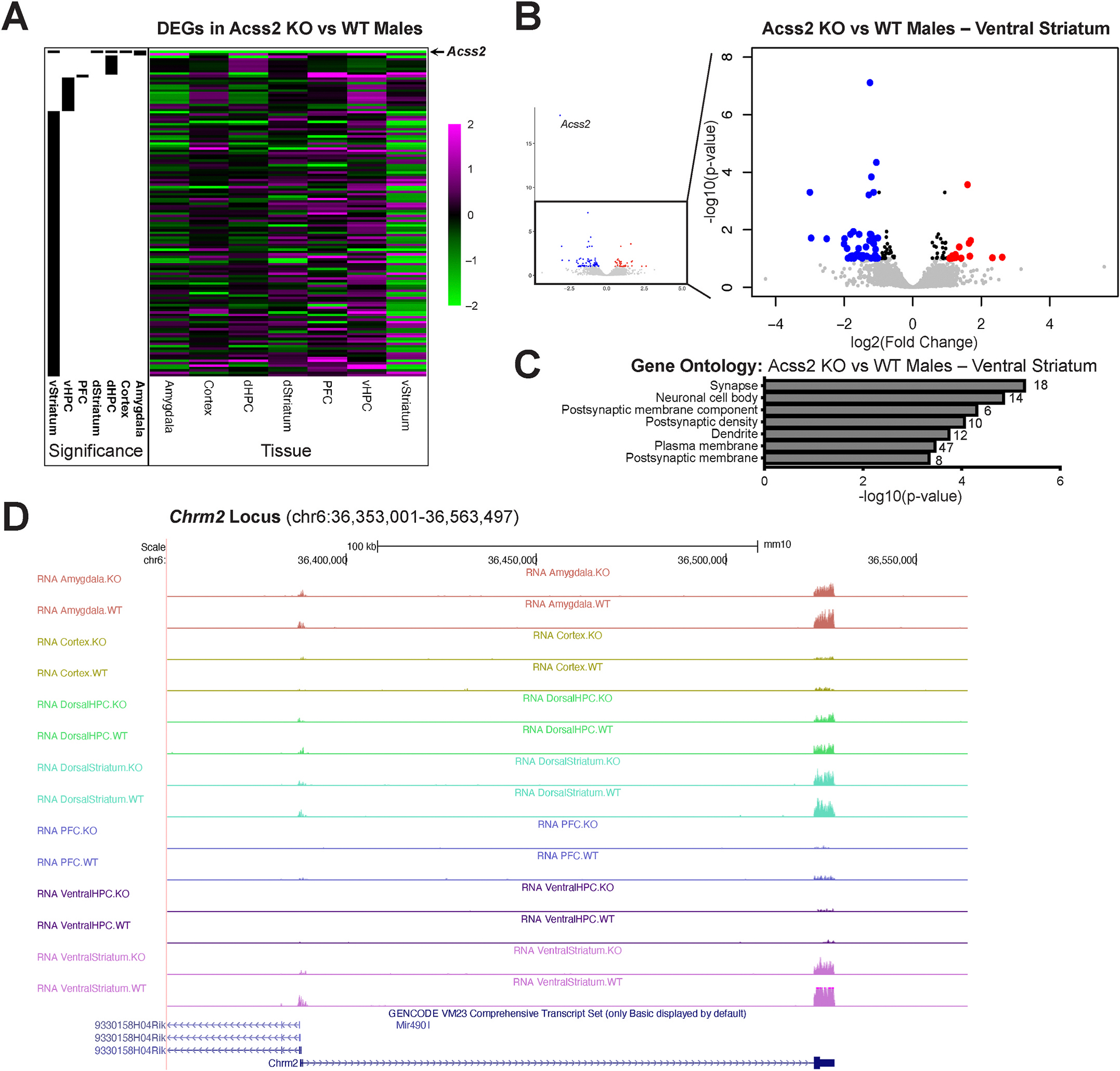
Transcriptional profiling across key brain regions following voluntary alcohol intake in WT and Acss2 KO mice. (A) Heatmap showing differentially expressed genes (DEGs, n = 135) across seven brain regions investigated in Acss2 KO vs. WT male mice following voluntary alcohol consumption. Black bars (left) denote significant DEGs corresponding to each region (FDR<0.1). Magenta (2) indicates increased expression in Acss2 KO vs WT males, black (0) indicates no differential expression between Acss2 KO vs WT males, and green (−2) indicates decreased expression in Acss2 KO vs WT males. (B) Volcano plot of ventral striatal DEGs (n = 111) identified between Acss2 KO vs WT males. Each dot represents a gene. Red dots denote significantly upregulated genes, and blue dots denote significantly downregulated genes. (C) Gene Ontology (GO) analysis of ventral striatal DEGs between Acss2 KO vs WT males showing enrichment of genes linked to synaptic functions. (D) Gene expression tracks of the *Chrm2* locus (chr6:36,353,001–36,563,497) across brain regions in male mice, showing specific significant decrease of *Chrm2* expression in the ventral striatum. DEGs, differentially expressed genes; dHPC, dorsal hippocampus; dStriatum, dorsal striatum; PFC, prefrontal cortex; vHPC, ventral hippocampus; vStriatum, ventral striatum; GO, Gene Ontology. (For interpretation of the references to color in this figure legend, the reader is referred to the Web version of this article.)

Next, we performed RNA-seq to profile transcriptional changes that accompany voluntary alcohol consumption across various key brain regions linked to AUD. We sacrificed mice following the final, 4-h drinking session and queried gene expression in male and female WT and Acss2 KO mice across the brain, focusing on regions implicated in alcohol use disorder (Koob and Volkow, 2010). These included the ventral striatum (which mediates alcohol reward), dorsal striatum, ventral and dorsal hippocampus, cortex, and amygdala. As expected, our principal component analysis revealed marked variance of gene expression across these brain regions investigated in both male and female animals (Supplementary Figs. 2A and B). In all brain regions and in both sexes, *Acss2* was the most significantly downregulated, with clear average loss of expression across all tissues (males shown in Fig. 2A and Supplementary Fig. 2E, females shown in Supplementary Fig. 2C).

In males, we found the highest number of differentially expressed genes (DEGs, n = 111) in the ventral striatum (Fig. 2A and Supplementary Table 1). Other brain regions showed considerably fewer DEGs between Acss2 KO and WT: 14 DEGs in the ventral hippocampus, 9 DEGs in the dorsal hippocampus, 2 DEGs in the amygdala, and 1 DEG each (*Acss2*) in the dorsal striatum, cortex and prefrontal cortex (Supplementary Table 1). In the ventral striatum, the majority (n = 71) of DEGs was significantly decreased in Acss2 KO animals compared to WT controls (Fig. 2B). Intriguingly, several of the ventral striatal DEGs were previously identified in human AUD patients, including downregulated *Akap12* (A-kinase anchoring protein 12), as well as upregulated *Camkk2* (*Ca/calmodulin-dependent protein kinase kinase 2*) and *Mef2C* (myocyte enhancer factor 2C)(Ho et al., 2022). Further, we found a significant enrichment of delayed primary response genes (PRGs; genes induced by neuronal activation independently of *de novo* protein translation, either in a rapid or delayed manner) and secondary response genes (SRGs; genes induced by neuronal activation dependent on translation of PRGs, in a delayed manner) (Tyssowski et al., 2018) among the ventral striatal DEGs (hypergeometric p = 0.000148). These included *Hcrtr2* (hypocretin receptor 2; Supplementary Fig. 3A), *Nap1l5* (nucleosome assembly protein 1-like 5; Supplementary Fig. 3B) and *Stac* (SH3 and cysteine rich domain; Supplementary Fig. 3C). The downregulation of primary and secondary response genes could indicate potential differences in alcohol-related plasticity in the ventral striatum of Acss2 KO mice compared to WT controls, which in turn might be contributing to the observed differences in drinking behavior (Fig. 1). This was further supported by Gene Ontology (GO) analysis, which revealed that, overall, downregulated ventral striatal DEGs were linked to synaptic functions, postsynaptic components and postsynaptic density/membrane (Fig. 2C). For example, we found decreased expression of delayed primary response gene *Chrm2*, a gene encoding for synaptic muscarinic acetylcholine receptor M2 (Fig. 2D). Chrm2 is strongly implicated in memory and cognitive function, and mutations of this gene were shown to predispose carriers to alcohol dependence and affective disorders (Luo et al., 2005).

By contrast, transcriptional changes between WT and Acss2 KO were more limited in female mice. This was in line with the less pronounced behavioral differences observed in females (Fig. 1). We found 69 DEGs in the prefrontal cortex, 12 DEGs in the dorsal hippocampus, 3 DEGs in the cortex, 2 DEGS each in the amygdala and ventral hippocampus, and 1 DEG each (*Acss2*) in the dorsal striatum and ventral striatum (Supplementary Table 1). The prefrontal cortical DEGs were enriched for genes related to myelination (Supplementary Fig. 2D), and only one of them (*Nrn1*) was a delayed response gene.

### 3.3. Depletion of H3K27ac underlies blunted gene expression programs in the ventral striatum of Acss2 KO mice following voluntary alcohol intake

Previously, we have shown that Acss2 drives alcohol-related histone acetylation in the dorsal hippocampus, leading to gene expression changes that underlie alcohol-associated learning (Mews et al., 2019). Whether Acss2-dependent histone acetylation plays a similar role in other brain regions that mediate other critical aspects of drinking behavior, however, remains unknown.

**Fig. 3.**
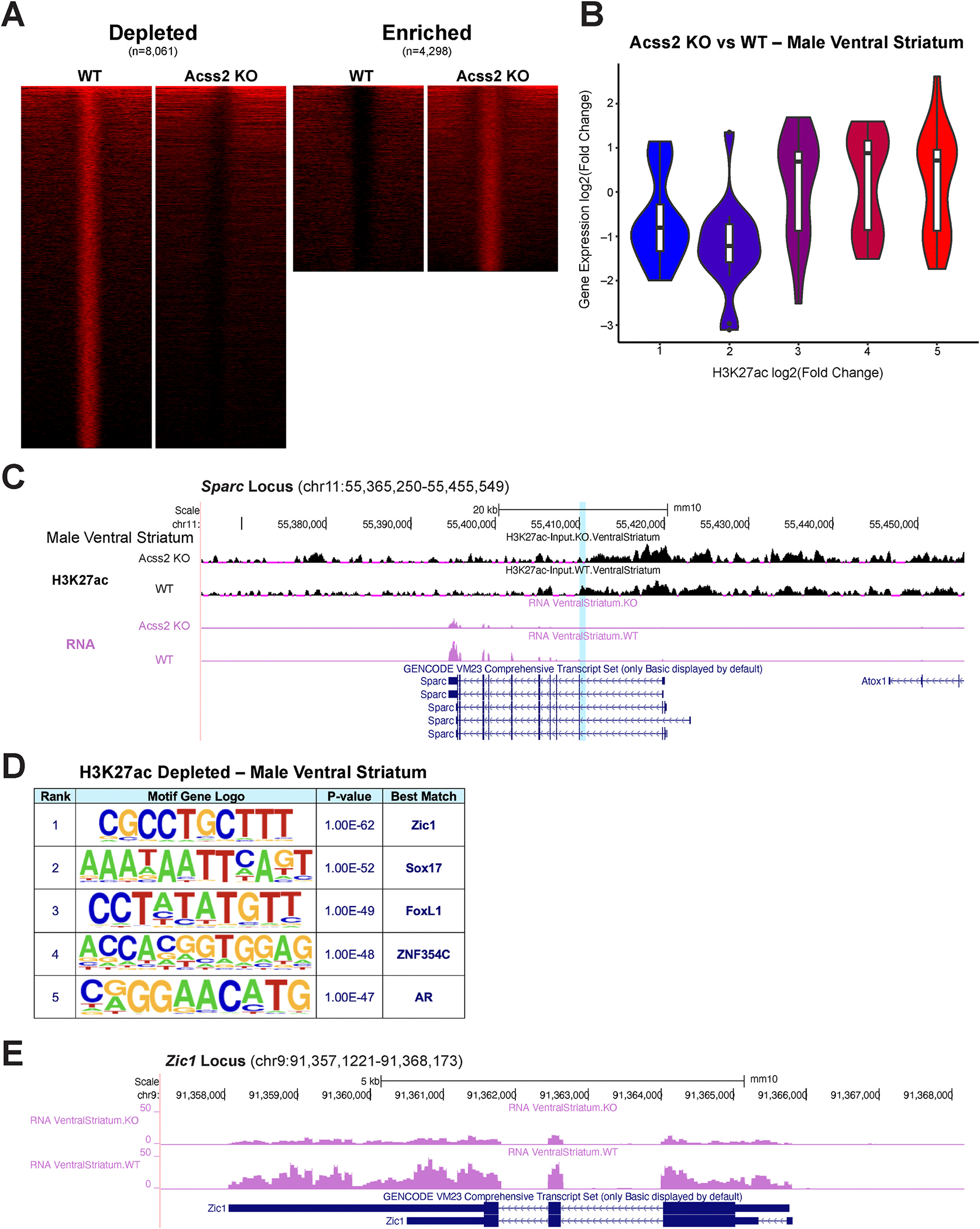
H3K27ac profiling of the ventral striatum following voluntary alcohol intake in male WT and Acss2 KO mice. (A) Heatmaps showing 2 kb windows of significantly depleted (n = 8061) and significantly enriched (n = 4298) H3K27ac peaks in the ventral striatum of Acss2 KO vs WT male mice following voluntary alcohol consumption. (B) Violin plots showing positive correlation between H3K27ac enrichment changes (ChIP-seq, x-axis) and gene expression changes (RNA-seq, y-axis) in the ventral striatum of Acss2 KO vs WT male mice following voluntary alcohol consumption. (C) Tracks of the *Sparc* locus (chr11:55,365,250–55,455,549) showing decreased H3K27ac enrichment (top two rows) and decreased gene expression (bottom two rows) in the Acss2 KO male ventral striatum compared to that of WT. (D) De novo motif analysis showing the top 5 significantly enriched transcription factor binding motifs in H3K27ac depleted peaks. (E) Gene expression tracks of the *Zic1* locus (chr9:91,357,1221–91,368,173).

To test this possibility in the ventral striatum, we next performed chromatin immunoprecipitation coupled with high throughput sequencing (ChIP-seq) targeting H3K27ac, a histone acetylation mark we have previously shown to be induced by alcohol-derived acetate (Mews et al., 2019). We focused on male mice given their more pronounced behavioral (Fig. 1) and transcriptional (Fig. 2) dysregulation described above. Comparing H3K27ac enrichment in the ventral striatum of WT and Acss2 KO mice following DID, we found that H3K27 acetylation was markedly decreased in animals not expressing Acss2 (Fig. 3A): we found 8061 significantly depleted peaks in Acss2 KO mice compared to WT mice, and only 4298 significantly enriched peaks (passing genome-wide statistical significance and exhibiting at least 4-fold change). We found a similar genomic distribution of depleted (Supplementary Fig. 4A) and enriched peaks (Supplementary Fig. 4B), with a slightly higher proportion of depleted peaks in intergenic space. Gene Ontology analysis revealed an enrichment of genes related to neuronal axonogenesis associated with depleted peaks, and genes related to synaptic membrane adhesion associated with enriched peaks (Supplementary Figs. 4C–D).

We next integrated our transcriptional and epigenetic datasets by comparing ventral striatal gene expression with H3K27ac enrichment (Supplementary Figs. 4E–G). As expected, genes with lower H3K27ac tended to show decreased transcription (Fig. 3B, with H3K27ac binning shown in Supplementary Fig. 4F), suggesting that the blunted drinking-related transcriptional states described above (Fig. 2) are related to the depletion of H3K27ac in the ventral striatum of Acss2 KO mice following drinking. For example, we found decreased H3K27ac at a putative enhancer (Fig. 3C, top two tracks, blue highlight) of the *Sparc* (Secreted protein acidic rich in cysteine) gene, the expression of which was also significantly decreased (Fig. 3C, bottom two tracks). Notably, *Sparc* was previously implicated in models of chronic intermittent alcohol exposure in the prefrontal cortex (Erickson et al., 2019).

We then performed *de novo* motif analysis of both lost and gained H3K27ac peaks in the ventral striatum and found that the binding motif of transcription factor Zic1 (zinc finger protein of the cerebellum 1) was the most enriched in H3K27ac peaks that were depleted in the Acss2 KO (Fig. 3D). Of note, the expression of Zic1 itself was significantly decreased in the ventral striatum (Fig. 3E), suggesting that this transcription factor might act as a regulator of alcohol-driven histone acetylation in this brain region.

## 4. Discussion

Here, we show that male mice not expressing Acss2 consume significantly less alcohol in a model of voluntary drinking. By performing transcriptional profiling across AUD-related brain regions, we find that decreased drinking is associated with blunted gene expression in the ventral striatum, a brain region closely linked to alcohol and drug reward (Borrego et al., 2022). We found that both behavioral and transcriptional differences between WT and Acss2 KO mice are more pronounced in male compared to female mice following voluntary alcohol intake. Further, we identified decreased acetylation of histone H3 lysine 27 (H3K27) as a potential epigenetic mechanism in the ventral striatum of male Acss2 KO mice.

Intriguingly, we found significantly decreased alcohol intake during the one bottle (1B) phase but not during the two-bottle choice (2BC) phase of the drinking-in-the-dark paradigm. This is surprising as drinking phenotypes generally show good correlation between the different versions of DID measuring alcohol intake and alcohol preference (Thiele and Navarro, 2014). One explanation for this differential effect is that Acss2 only mediates voluntary alcohol consumption in the absence of other liquids. However, as in our experiments the 2BC phase always preceded the 1B phase, it is also possible that the different behavioral outcomes were due to differences in alcohol drinking history. This would suggest that Acss2 plays a more important role in mediating long-term alcohol consumption compared to initial drinking. This possibility is further supported by our analysis of cumulative ethanol intake, which revealed a decreasing trend in Acss2 KO mice during the first (2BC) week of our DID paradigm (Fig. 1C), and reached statistical significance during the second (1B) week (Fig. 1F). Whether longer exposure to 2BC or switching the order of the 1B and 2BC phases would manifest in decreased alcohol preference and decreased drinking during the 2BC phase in Acss2 KO mice remains to be further explored.

Consistent with the literature, we observed greater levels of drinking in female compared to male mice (Rhodes et al., 2005). Intriguingly, the difference in ethanol consumption between Acss2 KO mice and WT controls were more pronounced in male compared to female mice in our experiment. This was accompanied by more severe transcriptional dysregulation, especially in the ventral striatum, suggesting that Acss2 may play an important role in mediating alcohol reward and consumption, especially in males. Surprisingly, we found that female Acss2 KO mice had significantly elevated blood ethanol concentrations (BEC) at the end of the DID paradigm compared to WTs, a difference we did not observe in males. As ethanol clearance was not affected by in Acss2 KO mice of either sex (Supplementary Fig. 1), the observed effects on histone acetylation and gene expression in males might potentially be confounded by the different amounts of ethanol consumed.

The overall levels of alcohol consumption were relatively low in our study across both sexes (Rhodes et al., 2007), and future experiments in genetic backgrounds selected for high drinking in the dark (Crabbe et al., 2011) will be informative to further outline the role of Acss2 during voluntary alcohol intake. Nevertheless, it is notable that Acss2 KO resulted in robust gene expression changes even for this relatively low level of drinking. In line with the more subtle behavioral effects, we found less pronounced transcriptional dysregulation in the brain of female Acss2 KO mice. One possibility that cannot be ruled out is that the dynamics of gene expression changes is different between males and females, and longer-term alcohol consumption could lead to more pronounced transcriptional dysregulation in females as well.

Intriguingly, while only 9 DEGs overlapped between male and female mice across all tissues, all of these genes have been previously implicated in AUD. In addition to *Acss2* (Ribeiro et al., 2017), we found changes in the expression of *Trf* (transferrin; carbohydrate deficient transferrin has been proposed as a biomarker of alcohol use (Solomons, 2012)) and *Atp1a1*, which is increased following prenatal exposure to alcohol (Zhou et al., 2011). Notably, six of the nine shared DEGs between males and females were strongly linked to myelination. *Mbp* (myelin basic protein), *Mag* (myelin-associated glycoprotein) and *Plp* (proteolipid protein) are decreased in the brain of human AUD patients (Lewohl et al., 2005). *Mal* (myelin and lymphocyte protein) expression is decreased in mice following binge drinking (Bent et al., 2022), and expression of *Kcnv1* (potassium voltage-gated channel modifier subfamily V member 1) correlates with voluntary ethanol consumption in both the cortex and the ventral striatum (Rinker et al., 2017). *Mobp* (myelin-associated oligodendrocyte basic protein) is decreased both in humans and animal models of alcohol use (Levey et al., 2014). *Acss2* itself is heavily implicated in lipid metabolism, both as a transcriptional regulator and as an important source of intracellular acetyl-CoA (Huang et al., 2018). While we have previously shown that this enzyme is primarily localized in the nucleus of differentiated neurons (Mews et al., 2017), it remains to be seen whether potential effects on cytoplasmic metabolism, lipid synthesis and myelinization also contribute to the behavioral differences observed in Acss2 KO mice, especially in the context of alcohol consumption.

An interesting category of genes enriched among the ventral striatal DEGs in males were delayed primary response genes or secondary response genes (Tyssowski et al., 2018). These are distinct groups of activity-regulated genes in neurons, which are induced in response to neuronal activation and have been linked to transcription-dependent synaptic plasticity, long-term potentiation, long-term depression, and synaptic scaling (Ahn et al., 1999; Ibata et al., 2008; Nguyen et al., 1994). Notably, several of the DEGs we identified in these categories have been previously implicated in alcohol use. For example, in humans, the *CHRM2* gene predisposes to alcohol dependence, drug dependence (Luo et al., 2005), as well as affective disorders including major depressive disorder (Wang et al., 2004). *Hcrtr2* encodes for the receptor of neuropeptide hypocretin/orexin, which is known to be dysregulated in alcohol dependence (Mahler et al., 2012) and compulsive alcohol drinking in mice (Aldridge et al., 2022). Further, Hcrt signaling in the amygdala was shown to be necessary for alcohol-seeking behavior in mouse models of alcohol dependence (Aldridge et al., 2022).

In line with blunted gene expression programs, we found that acetylation of H3K27 tended to be depleted in the ventral striatum of Acss2 KO male mice following alcohol drinking. We have previously shown that Acss2 mediates alcohol-driven histone acetylation in the brain by converting alcohol-derived acetate into acetyl-CoA, which is deposited on histones by histone acetyltransferases (Mews et al., 2019). Importantly, our current observations of decreased H3K27ac in Acss2 KO animals are in line with these findings. Our *de novo* motif analysis of depleted H3K27ac peaks identified transcription factor Zic1 as the most enriched binding site. Interestingly, mRNA levels of *Zic1* itself were significantly decreased in the KO mice. Zic1 has been previously implicated in brain development (Inoue et al., 2007) and Zic1-deficient mice show hypoplastic cerebellum and spinal cord (Aruga et al., 2002). Here, we identify a potential new role of Zic1 in the ventral striatum to mediate alcohol-related transcriptional adaptations that could underlie voluntary drinking. In future experiments, we will test whether inhibition or loss of Zic1 phenocopies Acss2 loss during drinking-in-the-dark or similar behavioral assays.

Taken together, our findings suggest that loss of Acss2 results in decreased voluntary alcohol intake, especially in male mice. We show that this behavioral phenotype is related to blunted alcohol-related gene expression in the ventral striatum compared to WT, driven by diminished histone acetylation in this brain region. Our findings further emphasize the critical role of Acss2 in alcohol metabolism and related epigenetic, transcriptional and behavioral changes, which warrant further exploration of this pathway as a potential future therapeutic target in humans suffering from alcohol use disorder.

## Supplementary Material

1

2

3

4

5

## Data Availability

Data will be made available on request.
