# Supplementary figures and images for: Decreased voluntary alcohol intake and ventral striatal epigenetic and transcriptional remodeling in male Acss2 KO mice
Source: Neuropharmacology. Author manuscript; Available in PMC 2025 Mar 1. (PMC11771284; doi:10.1016/j.neuropharm.2024.110258)

Supplementary Figure 1

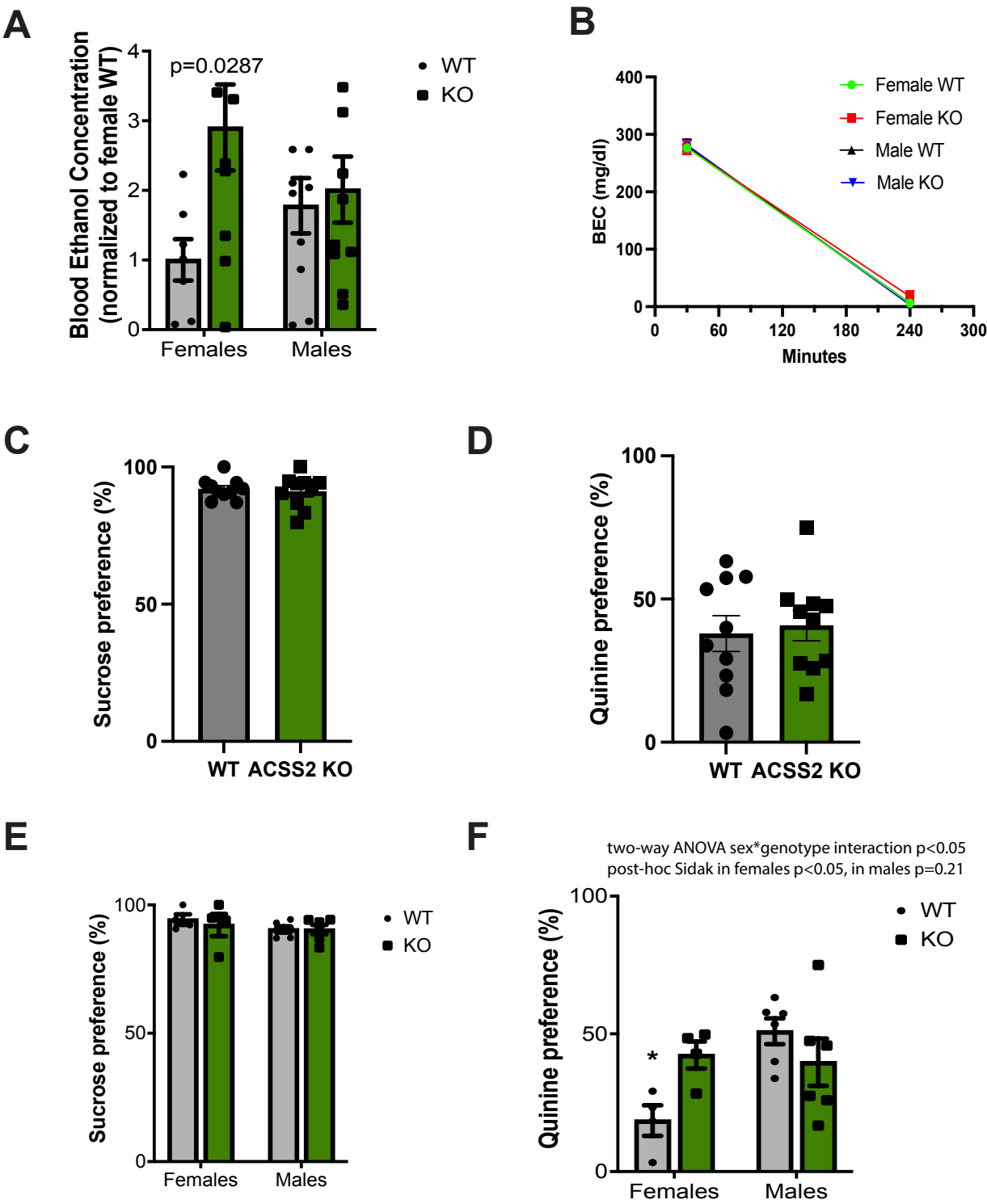

Supplement: 2 [file NIHMS2046390-supplement-2.pdf]

# Supplementary Figure 3

A

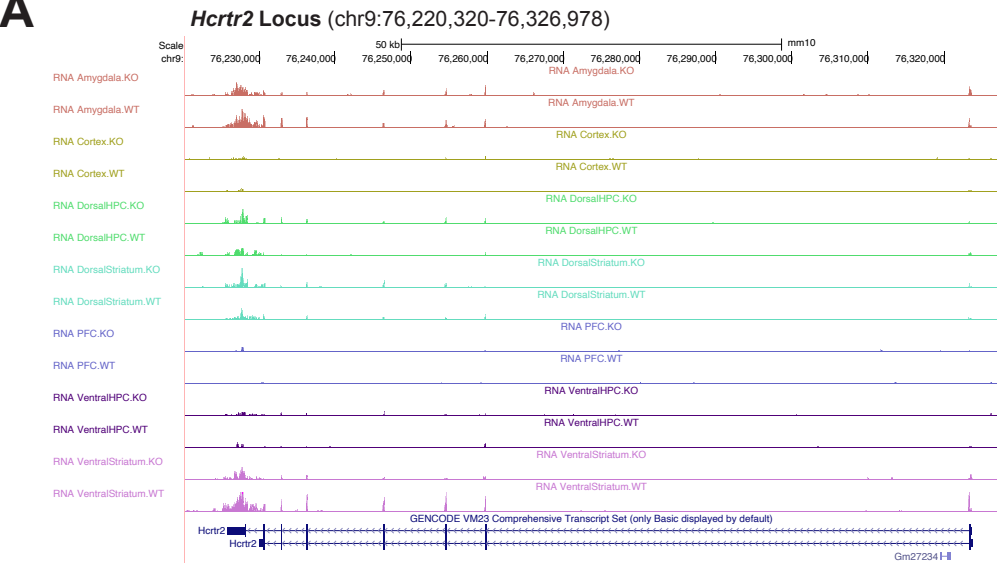

B

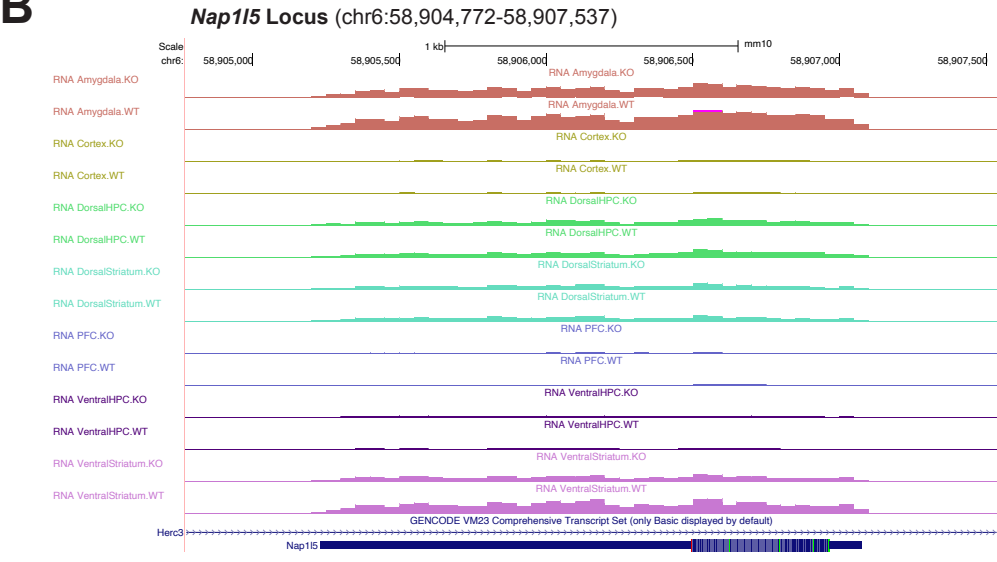

C

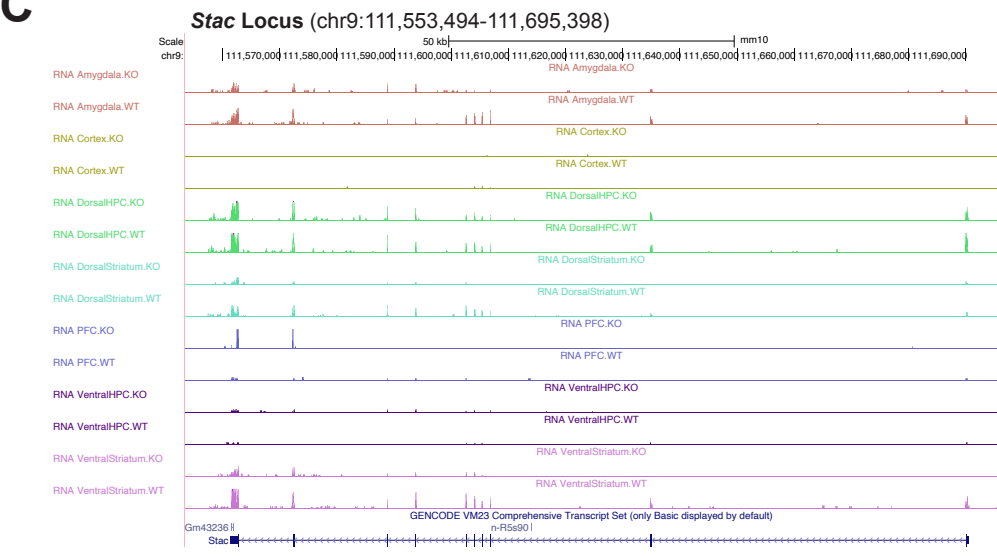

Supplement: 4 [file NIHMS2046390-supplement-4.pdf]
